# Gamma secretase orthologs are required for lysosomal activity and autophagic degradation in *Dictyostelium discoideum*, independent of PSEN (presenilin) proteolytic function

**DOI:** 10.1080/15548627.2019.1586245

**Published:** 2019-03-21

**Authors:** Devdutt Sharma, Grant Otto, Eleanor C. Warren, Philip Beesley, Jason S. King, Robin S. B. Williams

**Affiliations:** a School of Biological Sciences, Royal Holloway, University of London, Egham, UK; b Department of Biomedical Sciences, University of Sheffield, Sheffield, UK

**Keywords:** Alzheimer disease, autophagy, development, *Dictyostelium*, γ-secretase, lysosomal trafficking, presenilin

## Abstract

Mutations in the γ-secretase complex are strongly associated with familial Alzheimer disease. Both proteolytic and non-proteolytic functions for the γ-secretase complex have been previously described in mammalian model organisms, but their relative contributions to disease pathology remain unclear. Here, we dissect the roles of orthologs of the γ-secretase components in the model system *Dictyostelium*, focusing on endocytosis, lysosomal activity and autophagy. In this model, we show that the orthologs of PSEN (psenA and psenB), Ncstn (nicastrin) and Aph-1 (gamma-secretase subunit Aph-1), are necessary for optimal fluid-phase uptake by macropinocytosis and in multicellular development under basic pH conditions. Disruption of either psenA/B or Aph-1 proteins also leads to disrupted phagosomal proteolysis as well as decreased autophagosomal acidification and autophagic flux. This indicates a general defect in lysosomal trafficking and degradation, which we show leads to the accumulation of ubiquitinated protein aggregates in cells lacking psenA/B and Aph-1 proteins. Importantly, we find that all the endocytic defects observed in *Dictyostelium* PSEN ortholog mutants can be fully rescued by proteolytically inactive *Dictyostelium* psenB and human PSEN1 proteins. Our data therefore demonstrates an evolutionarily conserved non-proteolytic role for presenilin, and γ-secretase component orthologs, in maintaining *Dictyostelium* lysosomal trafficking and autophagy.

**Abbreviations:** Atg8: autophagy protein 8a; Aph-1: gamma-secretase subunit Aph-1; crtA: calreticulin; ER: endoplasmic reticulum; GFP: green fluorescent protein; GSK3B: glycogen synthase kinase 3 beta; Ncstn: nicastrin; PSEN1: presenilin 1; psenA and psenB: *Dictyostelium* presenilin A and B; TRITC; tetramethylrhodamine isothiocyanate.

## Introduction

Many studies have sought to explore a role for the γ-secretase complex (and PSEN [presenilin] proteins) in the pathology of Alzheimer disease [1–4]. One function of the mammalian complex (consisting of PSEN1 [presenilin 1], APH1 [aph-1 homolog, gamma-secretase subunit], NCSTN [nicastrin] and PSENEN/PEN2 [presenilin enhancer, gamma-secretase subunit]) or PSEN1 proteins alone is to regulate endocytosis, lysosomal acidification, and autophagy [5–7]. This role has been implicated in disease pathology, since mutations in PSEN1 proteins associated with familial Alzheimer disease result in elevated lysosomal pH, and aberrant autophagy in mouse models [7–9]. Dysfunctional endosomal-lysosomal and autophagic pathways have also been implicated in the pathogenesis of several other neurodegenerative disorders, including amyotrophic lateral sclerosis (ALS) and Parkinson disease [10–13]. These studies have given rise to a theory of neurodegenerative disease pathology relating to reduced protein clearance [13–15].

The role of the γ-secretase complex is primarily thought to be through its proteolytic activity and cleavage of target proteins [2–4,16]. Here, PSEN proteins contain two key aspartic acid residues necessary for the proteolytic activity of the complex in cleaving a range of substrates including the amyloid-β and NOTCH proteins [17]. However, the γ-secretase complex has also been proposed to act through non-proteolytic scaffolding functions in a variety of model organisms [18–24]. In mammalian models, these functions include stabilizing the binding of CTNNB1 and GSK3β, where Alzheimer disease causing mutations result in reduced stability of CTNNB1 [18,19,25,26], and in endoplasmic reticulum (ER) calcium regulation [27,28]. In *D. melanogaster*, presenilin proteins have been found to modulate the levels of CREBBP (cyclic-AMP Response Element Binding protein) independently of proteolytic activity [29]. A similar non-proteolytic function for presenilin proteins or the γ‐secretase complex has also been proposed in both *C. elegans* [30] and the moss *P. patens* [20]. However, although protease-independent functions of the γ-secretase complex have been observed in evolutionarily diverse species, our understanding of the mechanistic significance of these functions and relevance to Alzheimer pathology remains limited.

To better understand the role of the γ-secretase complex, several studies have employed the social amoeba *Dictyostelium discoideum* [22,31]. *Dictyostelium* contains orthologs of the core components of the complex including two PSEN (presenilin) proteins (psenA and B), Aph-1 and Ncstn [22,23,31]. In *Dictyostelium*, the complex components play roles in multicellular development, in cyclic AMP signalling and in intracellular calcium release [22] as well as in phagocytosis [31]. Although proteolytic targets(s) for the complex in *Dictyostelium* have not been defined, *Dictyostelium* psenA and psenB proteins show proteolytic activity in a Notch reporter assay [22]. In multicellular development, psenB plays a non-proteolytic (scaffold) function, since removal of the two key catalytic aspartic acids (348 and 394) does not block the formation of mature fruiting bodies. Furthermore, the roles of psenA and psenB proteins in *Dictyostelium* development are also complemented using the proteolytically inactive human PSEN1 protein, lacking the key catalytic aspartic acids 257 and 385 [22]. These studies have demonstrated the relevance of using *Dictyostelium* to examine the cellular and developmental roles of presenilin proteins and orthologs of other γ-secretase components.

*Dictyostelium* has been widely used as a model organism in a range of cell and molecular studies, often enabling translation to mammalian models [32,33]. It has been used in the study of endosomal- and autophagy-lysosomal systems and related pathways where WASH is required for lysosomal recycling and both autophagic and phagocytic digestion [34]; mucolipin is required for lysosomal exocytosis [35]; myosin I is involved in membrane recycling from early endosomes [36] as well as many other discoveries [37–41]. Here we advance our understanding of the role of the *Dictyostelium* orthologs of γ-secretase complex components in endocytosis and autophagy by demonstrating that these components are required for the efficient activity of these processes. We further show that regulation of endocytosis and autophagy activities occurs through a proteolysis-independent mechanism, where these activities are conserved in the human PSEN1 protein. We also show that loss of these proteolysis-independent functions leads to an increase in large poly-ubiquitinated autophagosome-like vesicles, demonstrating an ancient and conserved non-proteolytic role of presenilin and orthologs of other components of the γ-secretase complex in proteostasis.

## Results

### *Dictyostelium* mutants lacking γ-secretase component orthologs are unable to endocytose efficiently

**Figure 1.**
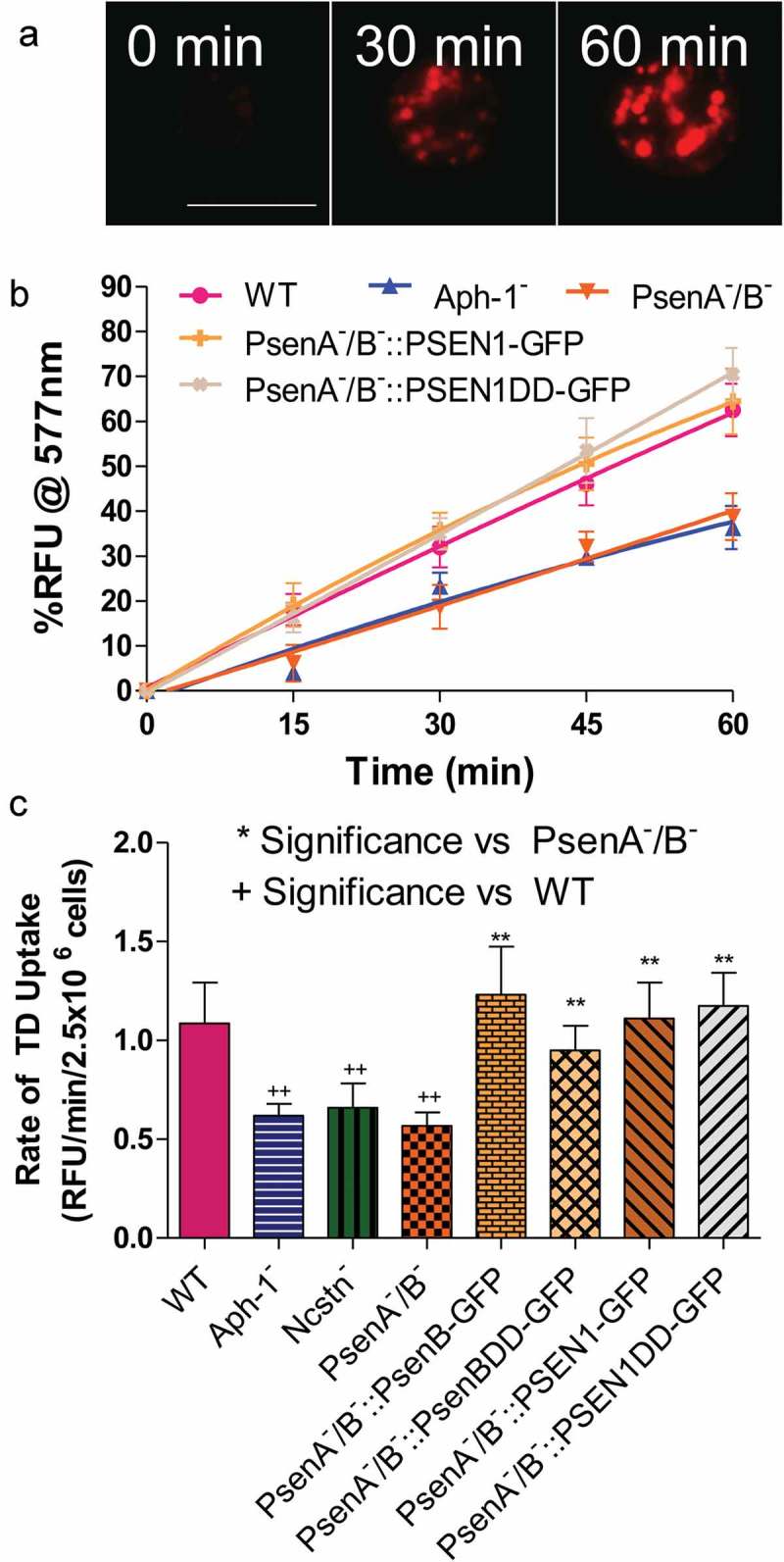
*Dictyostelium* mutants lacking γ-secretase component orthologs are unable to endocytose at wild type levels. *Dictyostelium* cells were shaken in media containing fluorescent TRITC-Dextran, and fluorescence uptake was used to monitor macropinocytosis over time, in wild-type cells, PsenA^−^/B^−^, Aph-1^−^ and Ncstn^−^ cells, and PsenA^−^/B^−^ following rescue by the proteolytic and non-proteolytic human PSEN1 proteins (PsenA^−^/B^−^::PSEN1-GFP and PsenA^−^/B^−^::PSEN1DD). (**a**) Representative images showing uptake of TRITC-Dextran by wild-type cells over a 60-min period. Scale bar: 10μ m. (**b**) Quantification of macropinocytosis over 60 min (±SEM). (**c**) Rate of macropinocytosis over 60 min (±SD). Data are provided from at least three independent experiments with technical duplicates. ^++^p > 0.01 to wild type, **p > 0.01 to PsenA^−^/B^−^.

Since the γ-secretase complex has been implicated in endocytosis in mammalian model organisms [5,6,42], we initially set out to investigate if orthologous components of this complex also play roles in *Dictyostelium* macropinocytosis – the dominant form of endocytosis and fluid-phase uptake in this organism. In wild-type cells, uptake of TRITC-Dextran was linear for 60 minutes and was therefore used to determine the rate of macropinocytosis (Figure 1(a,b), Figure S1). Macropinocytosis in the absence of the γ-secretase complex orthologs was analysed through the use of stable isogenic cells lacking Ncstn [22], Aph-1 or both presenilin proteins (psenA/B [22]). We observed that loss of all three γ-secretase component orthologs caused a 40–50% decrease in fluid-phase uptake (Figure 1(c)), and this effect was not due to altered exocytosis (Figure S2). These data suggest a role for the three γ-secretase component orthologs in regulating endocytosis in *Dictyostelium*.

A previous study has suggested that the *Dictyostelium* γ-secretase complex shows both proteolytic and non-proteolytic functions of psenB [22], thus we sought to distinguish these roles in macropinocytosis. In these experiments, the *Dictyostelium* psenB-GFP protein was expressed in PsenA^−^/B^−^ cells, in addition to a mutated version of the protein lacking the two key aspartic acid residues necessary for proteolytic activity (psenBDD-GFP) [22]. In both cases, wild type macropinocytosis levels were restored (Figure 1(b,c)), suggesting that this role of psenB is through a non-proteolytic function. Furthermore, expression of the wild type (PSEN1-GFP) and proteolytically inactive (PSEN1DD-GFP) human PSEN1 protein also rescued the macropinocytosis defect in PsenA^−^/B^−^ cells (Figure 1(b,c), Figure S1) demonstrating the evolutionary conservation of this function, although expression of proteolytically active PSEN1-GFP did not restore the macropinocytosis defect in cells lacking Ncstn (Figure S3). These data suggest that, in *Dictyostelium*, macropinocytosis was dependent upon Aph-1, Ncstn and a non-proteolytic function of psenB.

### *Dictyostelium* mutants lacking γ-secretase component orthologs show pH-dependent development

**Figure 2.**
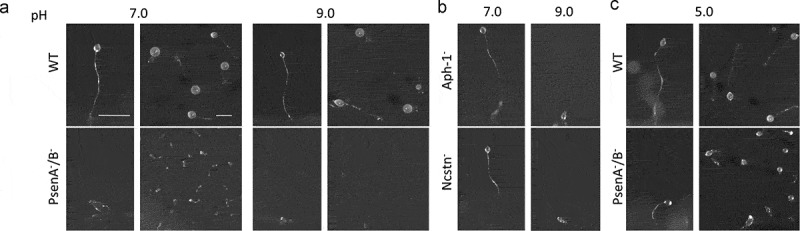
*Dictyostelium* mutants lacking γ-secretase component orthologs are unable to development under varying pH conditions. The development of *Dictyostelium*, through starvation over a 24-h period on nitrocellulose filters, leads to the formation of fruiting bodies consisting of round spore heads held aloft by a stalk, and provides a qualitative approach to monitor development in mutants. (**a**) At pH 7 and pH 9, wild type cells are capable of normal multi-cellular development forming fruiting bodies. In contrast, PsenA^−^/B^−^ cells are unable to develop at pH 7, forming short variable structures, and development is further inhibited at pH 9 where cells are unable to aggregate. (**b**) Aph-1^−^ and Ncstn^−^ cells form wild-type fruiting bodies at pH 7 but development is blocked at pH 9 (see also **Figure S4**). (**c**) Under acidic conditions, pH 5, wild-type cells form morphologically normal fruiting bodies, and PsenA^−^/B^−^ development is partially rescued, showing the formation of small fruiting bodies with round spore heads and stalks, similar to that shown for wild-type cells. Images are representative of triplicate experiments. Scale bar: 1 mm.

Since nutrients ingested in macropinosomes are degraded by fusion with lysosomes [43], and deficiencies in acidification decrease endocytic rate [44] we next investigated a role for orthologs of γ-secretase complex components in lysosomal acidification. For these experiments, we initially employed a qualitative development assay, where *Dictyostelium* mutants with severe acidification defects are unable to form mature fruiting bodies in neutral pH conditions [45]. We therefore tested the ability of mutants lacking orthologs of γ-secretase complex components to develop over 24 hours under neutral (pH 7) and basic conditions (pH 9). In both conditions, wild type cells were able to develop into mature fruiting bodies consisting of a basal disk, a stalk, and a spore head. However, while Ncstn^−^ and Aph-1^−^ cells were able to form fruiting bodies under neutral conditions, both failed to develop at pH 9 (Figure 2(b), Figure S4), consistent with defective lysosomal acidification. As reported previously [22], PsenA^−^/B^−^ cells were unable to develop under neutral conditions showing more severe developmental defects than Ncstn^−^ or Aph-1^−^ cells, halting at the mound stage, but development was reduced further under basic conditions at pH 9 (Figure 2(a)).

We then speculated that if the block in development of PsenA^−^/B^−^ cells at neutral pH was related to defects in lysosomal acidification, development may be restored by simply reducing the extracellular pH. We therefore examined development of PsenA^−^/B^−^ cells at pH 5 [22,45]. While acidic conditions had no observable effects on the development of wild type cells the development of PsenA^−^/B^−^ cells was partially rescued, forming morphologically normal but smaller sized fruiting bodies (Figure 2(c)). This rescue suggested that the developmental defects observed are at least partly due to defects in pH homeostasis, consistent with a role for the *Dictyostelium* orthologs of γ-secretase complex components in lysosomal acidification. However, the differences in the developmental phenotype between PsenA^−^/B^−^ cells and Ncstn*^−^* or Aph-1^−^ mutants indicate that psenA and psenB proteins may also function through other independent roles in *Dictyostelium* development.

Importantly, the development of PsenA^−^/B^−^ cells was fully rescued by expression of either proteolytically active or inactive *Dictyostelium* psenB or human PSEN1 proteins (Figure S5) [22] indicating a non-proteolytic role for these proteins in pH regulation.

### *Dictyostelium* mutants lacking orthologs of γ-secretase components show defective lysosomal degradation

**Figure 3.**
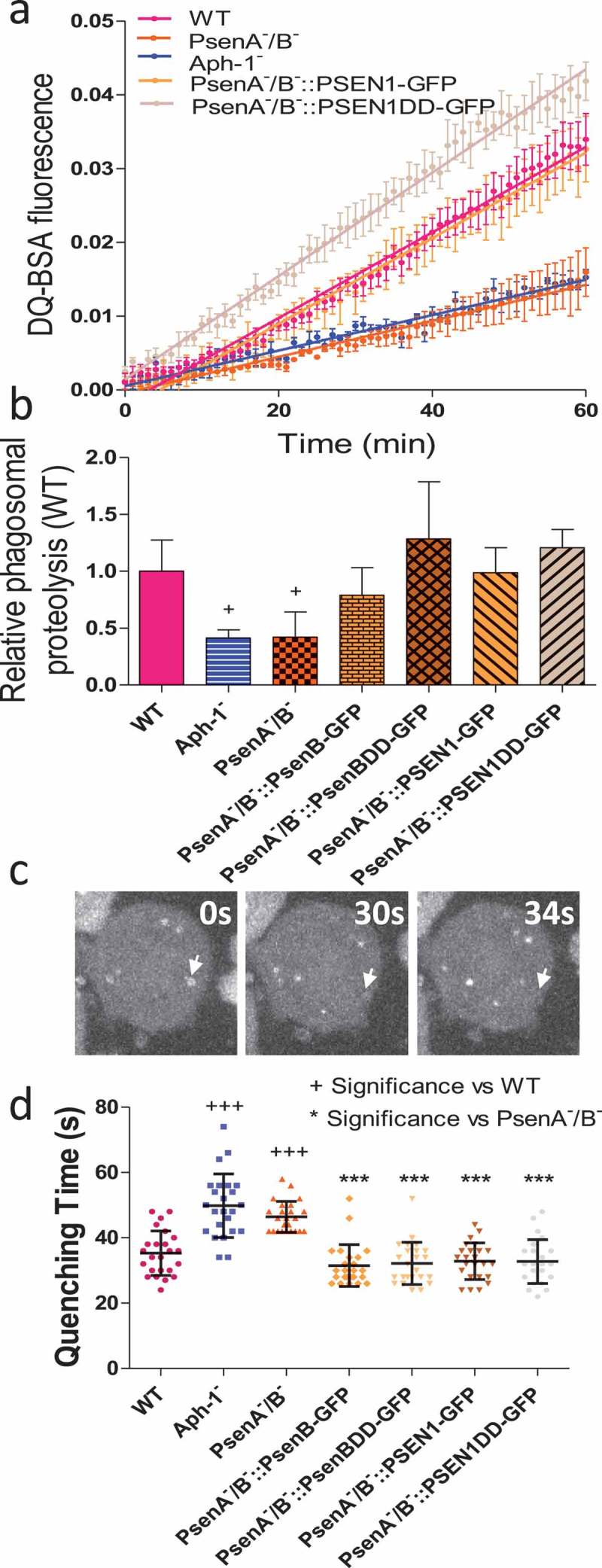
*Dictyostelium* mutants lacking γ-secretase component orthologs show abnormal lysosomal activity. Phagosome degradation is quantified by monitoring the increase in fluorescence of DQ-BSA-coated beads, taken up by phagocytosis, which becomes unquenched upon hydrolysis. This approach was used to assess in wild type cells, PsenA^−^/B^−^, and Aph-1^−^ cells, and PsenA^−^/B^−^ cells following rescue by the proteolytic and non-proteolytic *Dictyostelium* psenB or the equivalent human PSEN1 proteins (PsenB-GFP and PsenBDD-GFP or PSEN1-GFP and or PSEN1DD-GFP) respectively. (**a**) Quantification of proteolysis shows loss of a functional γ-secretase complex reduces phagolysosomal degradation, and this is restored by proteolytically active or inactive human PSEN1, from quadruplicate independent experiments (±SEM), which is reflected in (**b**) the rate of lysosomal acidification in (±SD). +P < 0.05. (**c**) *Dictyostelium* cells exhibit puncta of GFP-Atg8 that may be tracked (arrow) over time to determine the quenching time of GFP due to acidification. (**d**) Quantification of GFP-Atg8 quenching time shows an increase in the absence of a functional γ-secretase complex, and this is restored by both *Dictyostelium* and human presenilin proteins (both proteolytically active and inactive) (n = 25). +++p > 0.001 to wild type, ***p > 0.001 to PsenA^−^/B.

In order to directly test whether disruption of orthologs of the γ-secretase complex components leads to defects in lysosomal activity, we measured the ability of mutant cells lacking these components to degrade phagosomes. As a professional phagocyte, *Dictyostelium* cells readily engulf extracellular particles into endocytic vesicles which immediately fuse with lysosomes to be degraded. We therefore utilised a simple assay measuring the phagosomal proteolysis of beads coated with the self-quenching dye DQ-BSA, which becomes fluorescent upon proteolysis (Figure 3(a)) [46]. In this assay, we observed a significant decrease in proteolysis upon loss of multiple *Dictyostelium* orthologs of γ-secretase complex components. Ablation of Aph-1 or PsenA/B caused a reduction in proteolysis activity to 41.4%±4.1% (p = 0.05) and 42.1%+12.8% (p = 0.03) respectively relative to wild type cells (Figure 3(b)). Upon expression of proteolytically active, or inactive *Dictyostelium* psenB, relative proteolysis was restored to 78.9%±9.9% (p = 0.10) and 128.5%±25.1% (p = 0.36) and expression of active or inactive human PSEN1 resulted in a degradation rate of 98.8%±12.6% (p = 1.00) and 120.8%±7.9% (p = 0.35). The reduction in phagosomal proteolysis following loss of Aph-1 was not restored by expression of human PSEN1 (Figure S6). Although this assay does not distinguish between defective phagosome-lysosome fusion and decreased lysosomal activity, these data demonstrate that full degradative activity is dependent upon the presence of Aph-1, and psenB functioning through a non-proteolytic mechanism, and this activity is conserved in the human PSEN1 protein.

### Loss of γ-secretase components orthologs causes defective autophagy

The degradation of intracellular components by autophagy is also dependent on lysosomal activity. As autophagy is required for normal development of *Dictyostelium* [37] and has been heavily implicated in supressing the accumulation of mis-folded proteins characteristic of Alzheimer and several other neurodegenerative diseases [11,13,15,47,48], we next investigated the effects of loss of orthologs of γ-secretase components on autophagy.

We first measured autophagosome acidification and degradation in mutant lacking Aph-1 and psenA/B activity. In these experiments, we employed a fluorescent marker, GFP-Atg8, which becomes lipidated and incorporated into the membrane of nascent autophagosomes as they expand. Following completion, autophagosomes rapidly fuse with lysosomes and acidify – quenching the luminal GFP fluorescence, while the exterior GFP-Atg8 is removed [38,41]. In *Dictyostelium* cells this can be clearly observed by microscopy and by quantifying the time required for GFP quenching after autophagosome formation, where a rate of acidification can be defined for individual vesicles (Figure 3(c); MovieS1) [38,41]. In wild type cells, GFP-Atg8 quenched in 35.3 ± 6.8 seconds (Figure 3(d)). In contrast, Aph-1^−^ cells showed significantly increased acidification time to 49.8 ± 6.8 seconds (p < 0.001), and PsenA^−^/B^−^ cells also significantly increased quenching time to 46.4 ± 9.7 seconds (p < 0.001). The acidification defect in PsenA^−^/B^−^ cells was rescued upon expression of proteolytically active versions of *Dictyostelium* psenB (32.2 ± 4.7 seconds) as well as a proteolytically inactive version of the protein (31.5 ± 6.5 seconds) suggesting this effect was mediated by non-proteolytic functions of these proteins. The acidification defect in PsenA^−^/B^−^ cells was also rescued by both proteolytically active and inactive versions of human PSEN1 protein (32.8 ± 6.4 and 28.8 ± 5.6 seconds respectively). Therefore, the defects in lysosomal activity we observed upon disruption of Aph-1 and psenA/B proteins also impact on autophagosome maturation, suggesting an explanation for both the observed defects in *Dictyostelium* development as well as cell pathology in the absence of these proteins.

**Figure 4.**
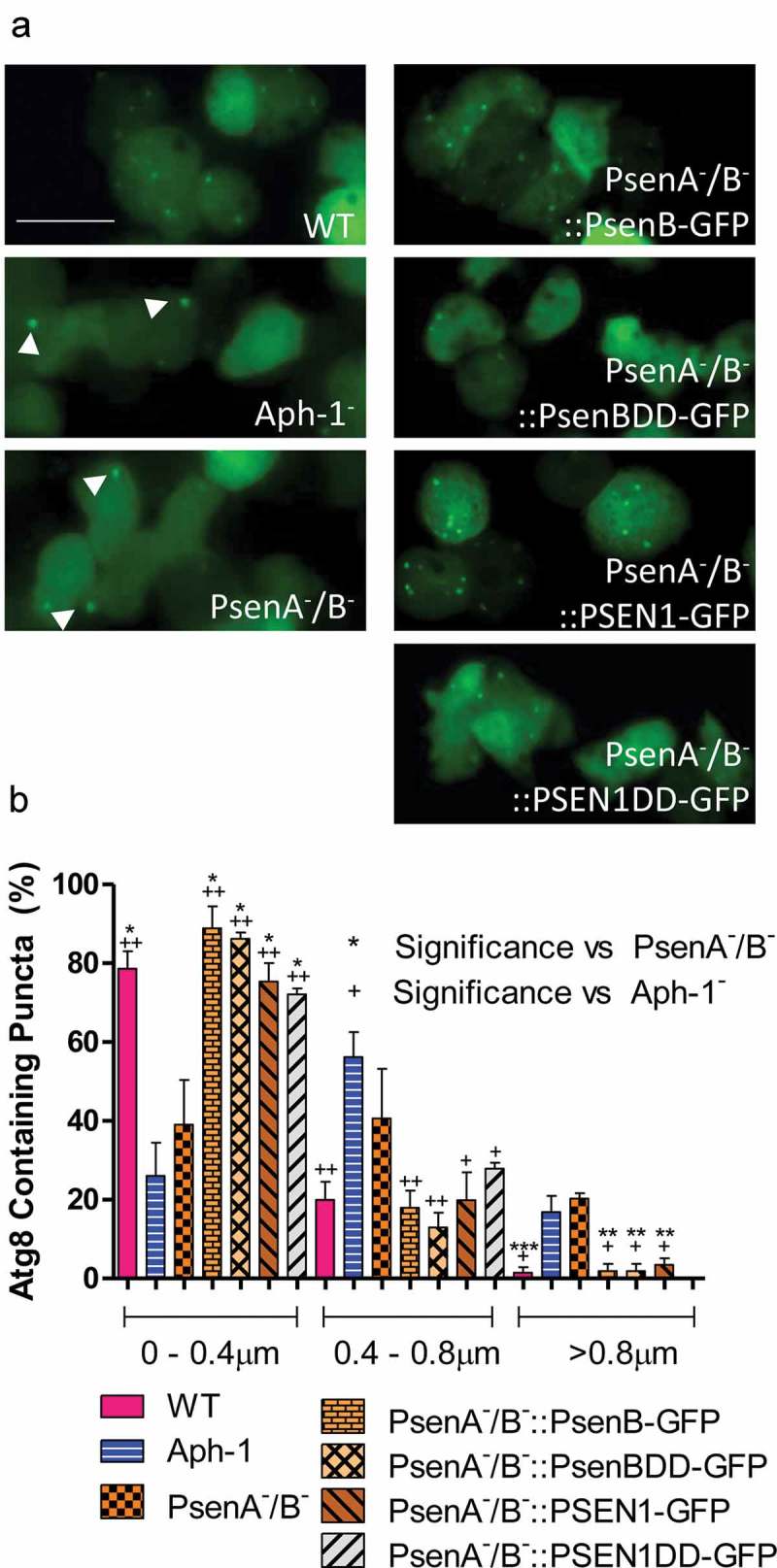
*Dictyostelium* mutants lacking γ-secretase component orthologs show aberrant size and localisation of GFP-Atg8. Visualization of GFP-Atg8 localisation in wild type cells, PsenA^−^/B^−^ and Aph-1^−^ and PsenA^−^/B^−^ cells following rescue by the proteolytic and non-proteolytic *Dictyostelium* psenB or the equivalent human PSEN1 proteins (PsenB-GFP and PsenBDD-GFP or PSEN1-GFP and or PSEN1DD-GFP) respectively. (**a**) Wild-type cells show multiple small and distributed GFP-Atg8-containing autophagosomes, whereas a single large punctum is seen in a proportion of PsenA^−^/B^−^ and Aph-1^−^ cells, that is no longer observed when cells are rescued following expression of *Dictyostelium* psenB orhuman PSEN1 (proteolytically active or inactive). Scale bar: 10μ m. (**b**) Quantification of the size of GFP-Atg8 -containing autophagosomes shows an increase in the absence of a functional γ-secretase complex, and this is restored by both *Dictyostelium* and human presenilin proteins (both proteolytically active and inactive). Data are derived from triplicate experiments measuring approximately 50 cells per experiment. ‘+’ compares to Aph-1^−^, ‘*’compares to PsenA^−^/B^−^, where * or + is P < 0.05, ** or ++ is P < 0.01, *** or +++ is P < 0.05.

Alterations in the process of vesicle acidification have been shown to regulate autophagy [15], in which cells recycle material for energy. In *Dictyostelium*, interruption in the autophagic pathway results in a decrease in the number and an increase in the size of GFP-Atg8 labelled structures [34,38,49]. As a result, we monitored their size and frequency in Aph-1^−^ and PsenA^−^/B^−^ cells for signs of autophagic dysfunction. Wild type cells expressing GFP-Atg8 show a majority of small (below 0.4μ m) diameter structures (79%), with very few structures above 0.8 μm (1.4%) representing normal functioning autophagosomes (Figure 4(a,b). In Aph-1^−^ cells, a significant reduction was seen in the occurrence of small structures to 26% (p < 0.01) and a significant increase in the formation of large GFP-Atg8-positive structures, to 17% (p < 0.05) typical of cells with reduced autophagy [37,39]. Similarly, PsenA^−^/B^−^ cells also showed single large GFP-Atg8 structures. These data suggest that loss of Aph-1 and psenA/B proteins caused autophagic dysfunction.

To test for the relevance of proteolytic activity in regulating autophagic function, we again expressed both active and inactive forms of both *Dictyostelium* psenB and human PSEN1 in PsenA^−^/B^−^ cells expressing GFP-Atg8. In these live-cell experiments, fluorescence was primarily derived from GFP-Atg8 (Figure S7), rather than fluorescently labelled psenB, Aph-1 and Ncstn proteins that localize to the ER (Figure S8). Expression of each form of presenilin rescued the ability of cells to form numerous small GFP-Atg8 structures, confirming this effect was related to loss of presenilin, again independent of proteolytic activity, and with conserved function shown in the human protein (Figure 4(a,b)).

**Figure 5.**
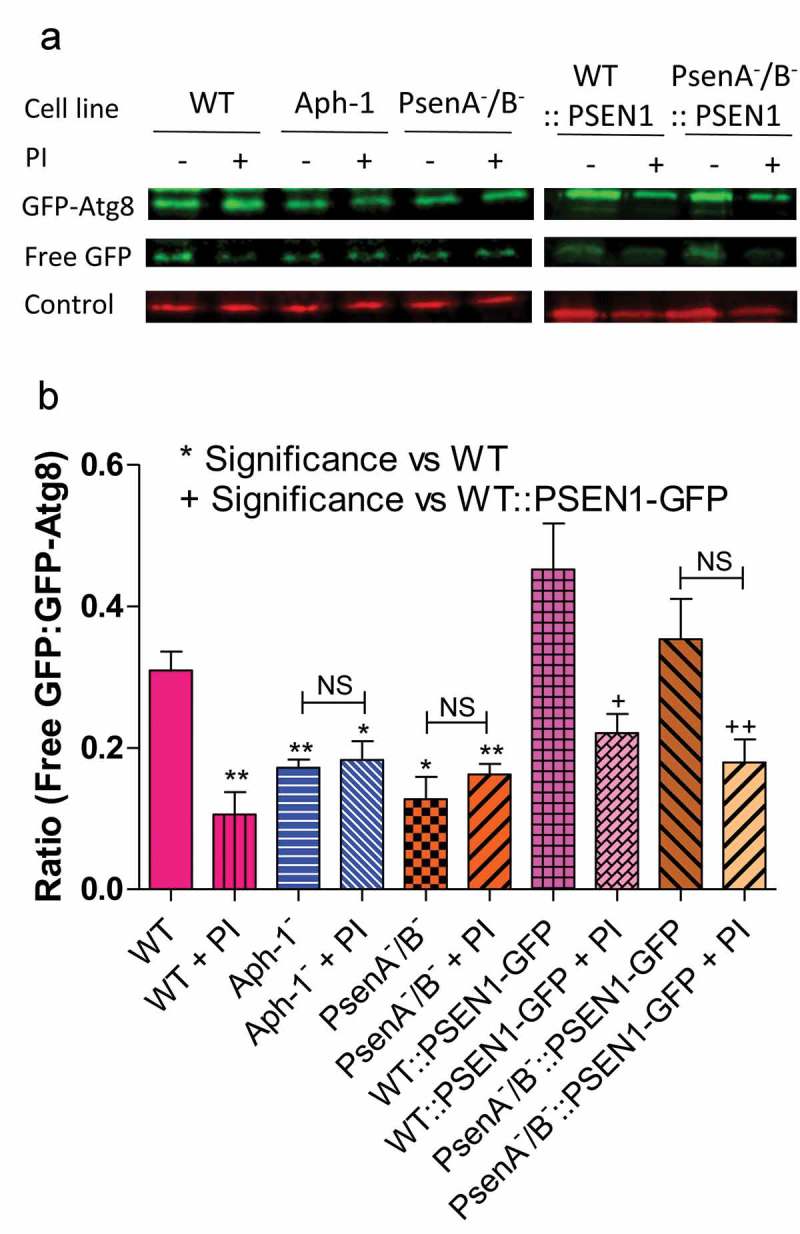
*Dictyostelium* mutants lacking γ-secretase component orthologs show decreased autophagic flux. Representative western blot analysis using an anti-GFP antibody enables comparison of levels of free GFP to GFP-Atg8 in the presence or absence of protease inhibitor (PI) treatment, allowing the comparison of wild type, Aph-1^−^, and PsenA^−^/B^−^ cell autophagic flux, and following rescue by the proteolytically active human PSEN1 protein (PSEN1-GFP). (**a**) Levels of free GFP are reduced in wild-type cells following PI treatment. In both Aph-1^−^ and PsenA^−^/B^−^ cells, free GFP levels are reduced in the absence of PI, and remain low following PI treatment. Expression of PSEN1-GFP in the PsenA^−^/B^−^ cells restores free GFP levels in untreated cells, and PI sensitivity. Endogenously biotinylated mitochondrial protein MCCC1 was used as a loading control. (**b**) Quantitation of free GFP to GFP-Atg8 ratios shows the absence of a functional γ-secretase complex reduces autophagic flux, and this is restored by the human PSEN1 protein. Data are derived from triplicate independent experiments (±SEM). ‘*’ compares to wild type, ‘+’ compares to PsenA^−^/B^−^, where * or + is P < 0.05, ** or ++ is P < 0.01; NS, not significant.

Finally, we investigated a role for the *Dictyostelium* γ-secretase component orthologs in autophagic degradation, by employing a western blot approach to examine autophagic flux as indicated by the cleavage of free GFP from GFP-Atg8 [50]. Comparing wild-type cells with PsenA^−^/B^−^ and Aph-1^−^ cells expressing GFP-Atg8, we measured the ratio of free GFP to total GFP-Atg8 in the absence and presence of lysosomal protease inhibitors as a measure of autophagic degradation. We showed that treatment of wild-type cells with protease inhibitor significantly inhibited the release of free GFP from GFP-Atg8 (p < 0.05) consistent with reduced autophagic degradation (Figure 5(a,b)). Ablation of psenA/B or Aph-1 resulted in a 39% and 49% decrease in the ratio of free GFP:GFP-Atg8, which was not significantly changed upon protease inhibitor treatment, suggesting a severe reduction in autophagic flux in PsenA^−^/B^−^ and Aph-1^−^ cells. Expression of the human PSEN1 in the PsenA^−^/B^−^ cells restored wild-type autophagic levels. These experiments confirm a role for psenA/B and Aph-1 proteins in autophagic regulation and demonstrate that this cellular function is conserved with the human PSEN1 protein.

### Loss of γ-secretase components orthologs results in large ubiquitin-positive structures

**Figure 6.**
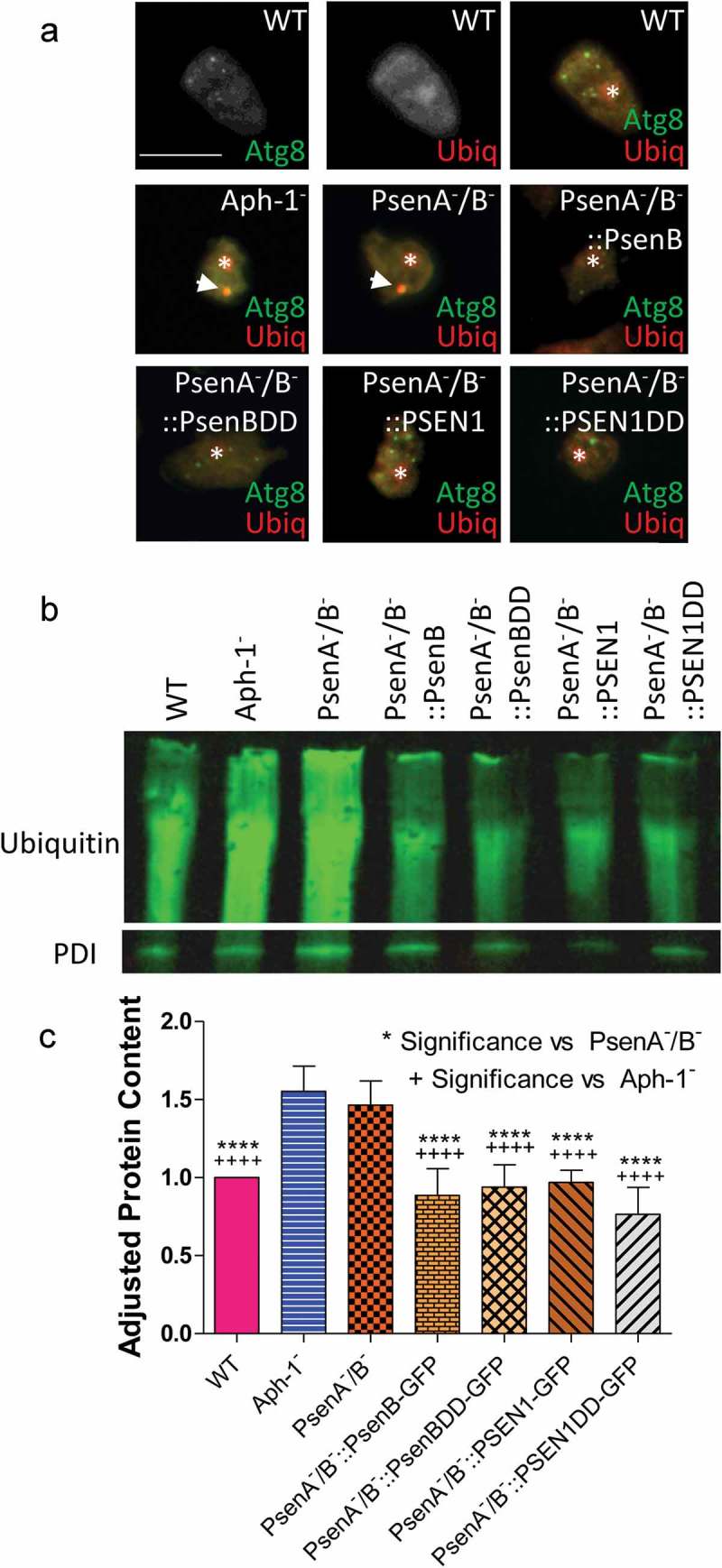
*Dictyostelium* mutants lacking γ-secretase component orthologs show ubiquitination defects. Analysis of ubiquitination levels in wild-type cells, PsenA^−^/B^−^ and Aph-1^−^ and PsenA^−^/B^−^ cells following rescue by the proteolytic and non-proteolytic *Dictyostelium* PsenB or the equivalent human PSEN1 proteins (PsenB-GFP and PsenBDD-GFP or PSEN1-GFP and PSEN1DD-GFP) respectively. (**a**) In cells expressing GFP-Atg8, immunofluorescence analysis shows co-localization of GFP and ubiquitin in single large puncta in Aph-1^−^ and PsenA^−^/B^−^ cells that are absent in wild-type cells, and are absent following rescue with the presenilin proteins. Scale bar: 10μ m. (**b**) Representative western blot analysis using anti-ubiquitin in the wild type, mutants and following rescue as indicated. Anti-PDI was used as a loading control for normalisation. (**c**) Quantification of anti-ubiquitin western blot shows a ~ 50% increase in large molecular weight ubiquitinated protein in cells lacking a functional γ-secretase complex when compared to normalized wild type levels. This increase is restored to wild type levels in PsenA^−^/B^−^ cells expressing *Dictyostelium* PsenB or human PSEN1 proteins regardless of proteolytic activity. Data are derived from 5 independent experiments.

In *Dictyostelium*, large GFP-Atg8-positive structures typically consist of high molecular weight ubiquitinated protein that cannot be cleared when autophagy is impaired [39]. We therefore examined the colocalization of GFP-Atg8 with ubiquitin-positive structures in the mutants lacking psenA/B and Aph-1 proteins. In wild type cells, the multiple small GFP-Atg8 puncta that were observed did not contain ubiquitinated proteins (Figure 6(a)). In contrast, the large GFP-Atg8-positive structures observed following loss of the γ-secretase complex, in both Aph-1^−^ and PsenA^−^/B^−^ cells, colocalized with ubiquitinated protein. Rescue of this phenotype in the PsenA^−^/B^−^ cells with both the proteolytically active and inactive version of the *Dictyostelium* PsenB and human PSEN1 proteins led to clearance of these ubiquitinated structures.

We then confirmed the effect of the loss of psenA/B and Aph-1 proteins on the accumulation of high molecular weight ubiquitinated proteins by western blotting (Figure 6(b,c)). Consistent with our results above, both the Aph-1^−^ and PsenA^−^/B^−^ cells showed a significant increase in high molecular weight ubiquitinated proteins (by 55% and 46% respectively; p = 0.0002 and p = 0.0001 respectively) again suggesting impaired protein degradation in these mutants. Accumulation of high molecular weight ubiquitinated protein was reversed by restoration of presenilin activity for both the *Dictyostelium* psenB (wild type and proteolytically inactive) and human PSEN1 (wild type and proteolytically inactive) rescue strains (Figure 6(b)). Importantly, accumulation of high molecular weight ubiquitinated protein indicated that these presenilin proteins and Aph-1 are physiologically important in maintaining basal autophagy levels and clearing misfolded proteins in *Dictyostelium*. Furthermore the function of these presenilin proteins are non-proteolytic and are conserved between *Dictyostelium* and humans.

## Discussion

In this study, we have shown that in *Dictyostelium*, orthologous components of the γ-secretase complex have roles in macropinocytosis, phagocytosis and autophagy, most likely through mediating lysosomal acidification. Recent studies in mammalian models of Alzheimer disease have investigated the role of these components in vesicular trafficking [7,11,13,15,48,51–53], and suggest that dysfunction of these processes may contribute to the progression and pathology of Alzheimer disease. These studies typically describe aberrant intracellular degradation as a result of dysfunction of presenilin proteins, rather than the entirety of the complex [5,42,51,52]. Here, we used *Dictyostelium discoideum* as a model to investigate orthologous components of the γ-secretase complex, taking advantage of the ability to delete individual genes in stable isogenic cultures to monitor changes in cell and developmental function, and to restore protein activity using wild type and proteolytically inactive presenilin proteins to rescue mutant phenotypes [22,31]. Using these approaches we show that in *Dictyostelium*, ablation of Ncstn, Aph-1 or both psenA/B genes results in deficient endocytosis and pH-dependent development, and loss of Aph-1 and psenA/B proteins gives rise to defects in lysosomal acidification and autophagy. We further show that these functions are dependent on the non-proteolytic activity of the psenA/B proteins that are conserved in the human PSEN1 protein.

We show that loss of Ncstn, Aph-1 or both psenA/B proteins inhibits macropinocytosis, a process by which cells take up large quantities of extracellular fluid. However, the exact mechanism that drives and controls the process is not yet fully understood [54]. *Dictyostelium* has proven to be an important model organism in macropinocytosis research and has led to a number of related discoveries, including the role of RasS in maintaining normal actin function in this process [55], the roles of phosphatidylinositol 3-kinases in macropinocytosis control [56], and the role of WASH in macropinosome recycling [49,54]. Further, both axeB/neurofibromin [57] and ndkC/nucleoside diphosphate kinase C [58] function to negatively regulate macropinocytosis. In mammalian cells, a number of these key regulatory pathways are conserved across evolution, including the function of RAS proteins, phosphatidylinositol 3-kinases, F-actin and WASH [49,59–61]. Our data now adds to this list, suggesting that orthologous components of the γ-secretase complex are necessary for optimal fluid uptake in *Dictyostelium* [55,57,62], involving a non-proteolytic activity of psenB protein, and these defects can be rescued by expression of a human PSEN1 protein. These results are consistent with but extend those shown in mammalian Alzheimer disease models, where loss of γ-secretase function results in aberrant macropinocytosis [5,6], and dysfunctional protein aggregate clearance for affected neuronal cells [5,6,42,52].

After extracellular material has been internalized, vesicles move through the intracellular endocytic pathway before finally fusing with lysosomes for degradation of vesicular material [63–65]. We show here, that *Dictyostelium* cells lacking Ncstn, Aph-1 or both psenA/B proteins fail to develop under basic pH conditions, consistent with that seen in other mutants that are unable to appropriately acidify their vesicles [45]. We then confirmed this role directly, by measuring autophagosome acidification, phagosome proteolysis [34,41] and autophagic flux in cells lacking Aph-1 or both psenA/B proteins. In mammalian models, dysfunctional lysosomal pH regulation has been linked to Alzheimer disease [66], in addition to other disease such as Pompe [67] and Niemann-Pick disease [68], as well as cholesterol uptake, and atherosclerosis – all resulting in inefficient degradation of intra-endosome material. In regard to Alzheimer disease, presenilin proteins have been implicated in a non-proteolytic, complex-independent role in maintaining lysosomal pH [15,51,52]. These studies suggest that presenilin regulates lysosomal pH through vacuolar-ATPase-mediated lysosomal acidification, resulting in altered calcium levels [51]. In *Dictyostelium*, we have shown that Ncstn, Aph-1 and psenA/B proteins are localized in the endoplasmic reticulum and this localization is unaltered in the absence of other complex components (Figure S8), consistent with that shown for Vmp1 involved with lysosomal function and autophagy [39] and a role of the complex in interacting with v-ATPase proteins for vesicle acidification and lysosomal activity. It is important to note here that our data do not explicitly link macropinocytosis and acidification defects, and further studies will need to investigate this in detail.

Previous research in *Dictyostelium* suggested that the γ-secretase complex is important for phagocytosis, and that this process is dependent upon proteolytic activity [31]. Our study confirms the role of Aph-1 and psenA/B proteins in phagocytosis, but demonstrates that it is not reliant upon proteolytic activity [22]. These data quantify the loss of (phago-)lysosomal proteolysis in cells lacking the orthologs of multiple γ-secretase complex components, and together with data on macropinocytosis, suggest that material ingested through both processes are unlikely to be efficiently degraded, and these effects are commonly observed in cellular models for Alzheimer disease [5,6,66].

Another cellular process dependent upon lysosomal degradation is autophagy [69]. *Dictyostelium* has been widely used to investigate autophagy and the importance of autophagy, and its role in multicellular development [40]. Here we show that cells lacking psenA/B or Aph-1 proteins share a number of phenotypes with autophagy dysfunctional mutants, including defective GFP-Atg8 localization [37,39], co-localisation between GFP-Atg8 and ubiquitin aggregates [39], and an abundance of high molecular weight ubiquitinated protein [39,70], reduced autophagic flux, and aberrant development. These data demonstrate that orthologs of multiple γ-secretase complex components play a key role in the autophagic process in *Dictyostelium*. Consistent with this, neurons of patients diagnosed with Alzheimer disease also accumulate neurotoxic peptides due to inefficient lysosomal acidification and a resultant build-up of autophagosomes rich in amyloid precursor protein [7,8,52]. We further show that, in *Dictyostelium*, this deficiency in autophagy and accumulation of ubiquitinated proteins is not caused by loss of psenB proteolytic activity, and this activity can be fulfilled by the human PSEN1 protein [7,51].

In this study, we demonstrate that the *Dictyostelium* orthologs of γ-secretase complex components play a key role in regulating macropinocytosis, phagocytosis, acidification of lysosomal vesicles, and autophagy. The reduction of these activities following the loss of two (psenA/B and Aph-1) or three (psenA/B, Aph-1 and Ncstn) orthologs of γ-secretase complex components suggest that these phenotypes may be regulated by a *Dictyostelium* γ-secretase complex, but we cannot explicitly demonstrate that these roles are not independent. Similarly, the interrelatedness of these cellular function is also consistent with a common role for a *Dictyostelium* γ-secretase complex in this process, and that this function is not dependent on the proteolytic activity of the putative γ-secretase complex. Finally, we demonstrate that these roles of the psenA/B proteins can be rescued in *Dictyostelium* through expression of human PSEN1. Thus we propose that, in *Dictyostelium*, an orthologous γ-secretase complex plays a key role in maintaining endocytosis, lysosomal trafficking and autophagy through a non-proteolytic function, conserved between *Dictyostelium* and humans across the vast evolutionary gap that separates these two organisms.

## Materials and methods

### Cell culture and maintenance

*Dictyostelium* wild type strain Ax2 were grown in HL5 medium (Formedium, HLB0103) supplemented with 10% glucose (Sigma-Aldrich, G8270). PsenA^−^/B^−^ cells and rescue strains, and Ncstn^−^ cells have been previously described [22], and are maintained in HL5 medium supplemented with/without 10 μg/ml hygromycin (Formedium, HYG1000).

### Plasmid construction and transformation

Aph-1^−^ mutant cells were generated by homologous integration using the Cre-Lox system [71].

Briefly, a knockout vector was designed consisting of a 5ʹ and 3ʹ arm of homology (primers:

GTG GAT CCT ATA AGT ATT TTA AAG ATT/AAC TGC AGA GAT ATT TAA AAA TGT TTC TTA CC and TTA TTC CAT GGA GTT TAT AAC GTT TT/AAT GGT ACC TTG ATA ATG TTA AAA TGA) in order to ablate a central 762 base pair region of the gene. The linearized vector was electroporated into wild-type *Dictyostelium* cells, and transformants were screened by PCR in order to identify homologous recombinants [72].

### Macropinocytosis and exocytosis assay

Rate of TRITC-Dextran uptake was measured as described previously [62]. Briefly, 2.5 × 10^7^ cells were resuspended in 5 ml of HL5 medium supplemented with 100 μl or 100 mg/ml TRITC-Dextran (Sigma-Aldirch, T1162). At time points 0, 15, 30, 45, 60, 90, and 120 min, 500 μl of this suspension was removed, washed in phosphate buffer and measured on a Perkin Elmer LS50B spectrophotometer. Relative change in fluorescence was calculated and normalised against total protein content of each respective cell line. The rate of uptake was measured using the linear function in GraphPad Prism.

To determine whether efflux was also affected by γ-secretase ablation a simple assay was utilised [73]. Briefly, 2.5 × 10^7^ cells were incubated in HL-5 with 100 mg/ml TRITC-Dextran for a period of 3 h. Following incubation, cells were washed and resuspended in 5 ml of phosphate buffer before shaking incubation. At time points 0, 15, 30, 45, 60, 90 and 120 min 500 μl of the suspension was removed and the fluorescence measured. Change in fluorescence was calculated relative to the measurement at time point 0 min of each respective cell line. The rate of efflux was calculated using GraphPad Prism.

### *Dictyostelium* development at varying pH

In these assays, 1 × 10^7^ cells were plated and allowed to develop on 47mm nitrocellulose filters (Millipore, HAWP04700) over 24 h [74]. Each filter was placed on an absorbent 3M paper pad (Millipore, AP1004700) soaked in phosphate buffer of varying pH. Phosphate buffer was prepared using varying amounts of KH_2_PO_4_ and K_2_HPO_4_ to modulate pH without altering ionic strength. Subsequent developmental phenotypes were imaged using a dissection microscope (Leica) and a QICAM FAST 1394 camera (QImaging).

### Fluorescence microscopy and quantification

Cells were imaged on an Olympus IX71 wide-field fluorescence microscope. Images were captured using a QICAM FAST 1394 camera. For measurement of Atg8-positive structure size cells were analysed using ImageJ [75] and cell size was measured across the largest diameter. More than 100 GFP-Atg8 positive structures were measured, using around 50 cells per experiment including at least three independent experimental repeats. For live-cell imaging, cells were imaged under a layer of ~1.5-mm thick 1% phosphate buffered agarose [49].

### Analysis of autophagosome maturation

The autophagy reporter GFP-Atg8 was expressed in cells using the extrachromosomal expression plasmid pDM430 [38]. To both reduce the movement of vesicles in the Z-plane and stimulate autophagosome formation cells were compressed under a thin layer of 1% agarose in HL-5 medium as previously described [38]. Cells were seeded in glass-bottomed microscopy dishes before removal of most of the medium, application of a ~ 2-mm thick agarose slab and compression by blotting with paper and capillary action. After 10 min, images were captured using a Perkin-Elmer Ultraview VoX inverted spinning disc microscope, using a 100 × 1.4NA objective and Hammamatsu C9100-50 EM-CCD camera. 4 Z planes at 1μM spacing were captured every 2 s. The point of autophagosome completion was determined by the characteristic enlargement and diming of GFP fluorescence [41]. The time until GFP-fluorescence was undetectable was subsequently measured from randomised, blinded movies captured from at least three independent experiments.

### Phagosomal proteolysis assays

Phagosomal proteolytic activity was measured by feeding cells DQgreen/Alexa Fluor 594 (DQ-BSA; Invitrogen, D12050) co-labelled 3-μm silica beads (Kisker Biotech, PSI-3.0COOH) as previously described [76]. Briefly 3 × 10^5^ cells/well were seeded in a 96-well plate before addition of beads, and fluorescence measured on a plate reader each minute in triplicate. Proteolysis was normalised to Alexa Fluor 594 fluorescence, over time to account for potential differences in bead uptake and rates normalized to wild-type cells to calculate relative activity.

### Western blotting and immunofluorescence

Analysis of high molecular weight ubiquitinated protein was carried out by western blot as previously described [70]. Briefly, the proteins of 2 × 10^5^ cells were separated by SDS gel electrophoresis and the membrane was probed with α-PDI [77] (an ER marker protein, disulfide isomerase a kind gift from Annette Muller-Taübenberger) as a loading control and α-ubiquitin (Cell Signalling Technology, P4D1) at 1:50, and 1:1000 dilutions, respectively. Relative amounts of high molecular weight ubiquitinated protein were calculated using Image Studio Lite (LI-COR Biosciences) and these were normalized against wild-type levels of ubiquitinated protein.

For colocalization of ubiquitin and GFP-Atg8, cells were fixed in −80°C methanol as previously described [78]. After fixation cells were probed with α-GFP (Chromotek, 3H9) and anti-ubiquitin (New England Biolabs, 3936S) antibodies at 1:500 and 1:200 dilution concentrations, secondary antibodies used were anti-rat Alexa Fluor 488 and anti-mouse Alexa Fluor 350 (Life Technologies, A-21,210 and A-31,552 respectively) at 1:1000 concentration dilutions. Cells were then imaged to visualise colocalization between ubiquitin and GFP-Atg8-positive structures.

To analyze autophagic flux a western blot based approach was utilised [50]. Briefly, 1.3 × 10^6^ cells were seeded in a 6 well plate before treatment with protease inhibitors (Roche Life Science, 05892791001) for 1 h, when cells were harvested and proteins extracted. Western blots were probed with an anti-GFP antibody (1:1000 dilution) and levels of the endogenously biotinyated mitochondrial protein MCCC1 detected with a streptavidin-conjugated antibody used as a loading control [79] (Thermo Fisher Scientific, S21378). The fluorescent ratio of free GFP to GFP-Atg8 was calculated to determine a measure of autophagic flux.

To visualize *Dictyostelium* γ-secretase components localization, cells were fixed using 80°C methanol [78] and probed with antibodies. Briefly, cells overexpressing GFP-tagged components were seeded onto coverslips and fixed by submerging in −80°C methanol for 30 min. Following fixation, cells were washed in room temperature phosphate-buffered saline, stained with anti-GFP and anti-crtA (calreticulin) antibodies (a kind gift from Annette Muller-Taübenberger), subsequently stained with appropriate secondary antibodies to allow for ER visualization, and nuclei were stained with DAPI.

## Statistical analysis

Statistical analysis depended upon the data analyzed. For normally distributed data a two-way ANOVA was utilised when comparing multiple data groups to each other. For data that is not normally distributed the Mann-Whitney test was used, and for data that was compared to a single normalised mean value a one sample T-test was used. Statistical analysis was carried out using GraphPad Prism Software.

## Data Availability

All data related to this study are available in the paper or via supplementary material.
