# Supplementary material for: Gamma secretase orthologs are required for lysosomal activity and autophagic degradation in Dictyostelium discoideum, independent of PSEN (presenilin) proteolytic function
Source: Autophagy. 2019 Mar 21;15(8):1407–18. doi: 10.1080/15548627.2019.1586245 (PMC6613883; doi:10.1080/15548627.2019.1586245)
Supplement: Supplemental Material [file kaup-15-08-1586245-s001.zip › 1586245_supplementary information/Supplementary figure and legends_changes accepted.docx]

**Sharma et al Supplementary figures**

**
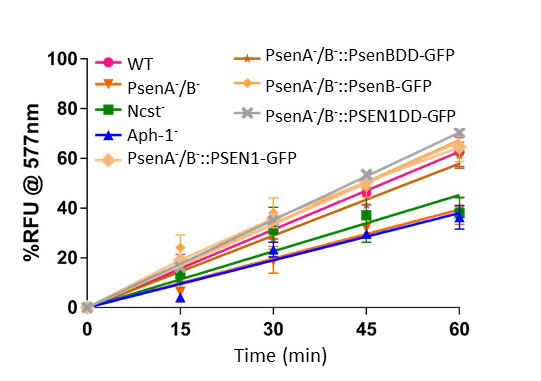
**

**Figure S1.** *Dictyostelium* macropinocytosis is dependent γ-secretase component orthologs in a non-proteolytic function. (**A**) Uptake of TRITC-Dextran over 120 min in wild-type, Aph-1^-^, PsenA^-^/B^-^, PsenA^-^/B^-^::PsenB-GFP, PsenA^-^/B^-^::PsenBDD-GFP, PsenA^-^/B^-^::PSEN1-GFP, and PsenA^-^/B^-^::PSEN1DD-GFP over 120 min. (**B**) Uptake of TRITC-Dextran over 60 min in wild-type, Aph-1^-^, PsenA^-^/B^-^, PsenA^-^/B^-^::psenB-GFP, PsenA^-^/B^-^, PsenA^-^/B^-^::psenBDD-GFP, PsenA^-^/B^-^, PsenA^-^/B^-^::PSEN1-GFP, and PsenA^-^/B^-^, PsenA^-^/B^-^::PSEN11DD-GFP over 120 min.


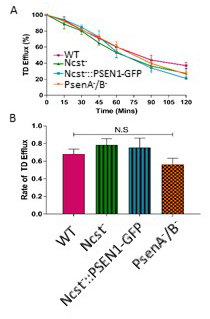


**Figure S2.** Exocytosis is unaffected by loss of γ-secretase component orthologs. (**A**) Exocytosis of TRITC-Dextran over 120 min from *Dictyostelium* wild-type, Ncstn^-^, Ncstn^-^::PSEN1-GFP and PsenA^-^/B^-^ cells. Exocytosis is expressed as a percentage of the value at 0 min after 180 min of TRITC-Dextran uptake (n=3). (**B**) The rate of TRITC-Dextran exocytosis exhibits no significant (p>0.05) difference between wild-type cells, Ncstn^-^, or PsenA^-^/B^-^ cells. Expression of PSEN1-GFP does not alter exocytosis rates.


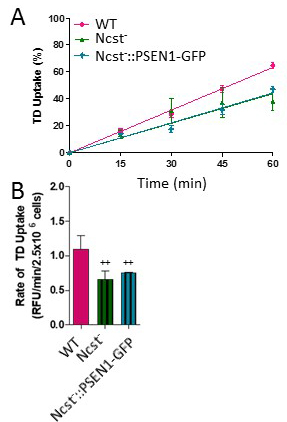


**Figure S3.** Reduction in *Dictyostelium* macropinocytosis by loss of Ncstn is not rescued by overexpression of Presenilin. (**A**) TRITC-Dextran uptake was measured over 120 min in wild-type, Ncstn^-^, and Ncstn^-^::PSEN1-GFP cells. Measurements were taken every 15 min for a 60-min period and background (0 min) was subtracted from subsequent readings (n=3). (**B**) Rate of TRITC-Dextran uptake was calculated and demonstrates no significant difference between Ncstn^-^ and Ncstn^-^::PSEN1-GFP cell lines, and ^++^ denotes p < 0.01 significance against wild-type cells.


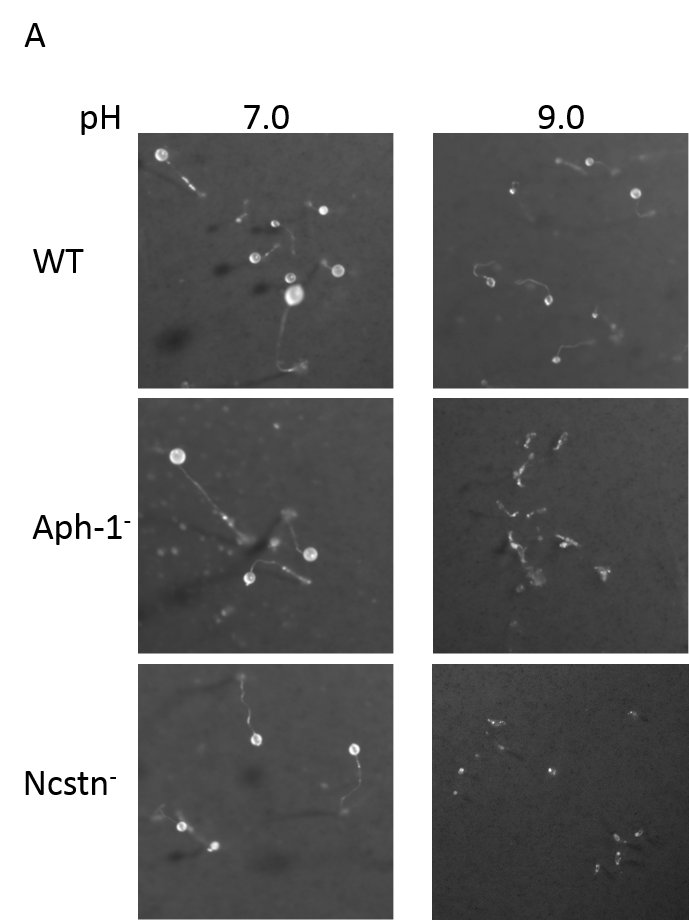


**Figure S4.** *Dictyostelium* Aph-1^-^ and Ncstn^-^ mutant development at pH 7 and pH 9. Wild-type *Dictyostelium* cells are able to develop normal fruiting bodies at pH 7 and pH 9. Aph-1^-^ and Ncstn^-^ null cells develop normal morphology fruiting bodies at pH 7, but are inhibited at pH 9 over 24 h. Data are representative of 3 independent experiments.

**
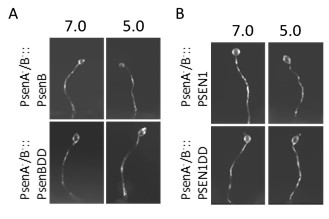
**

**Figure S5.** Development of PsenA^-^/B^-^ rescue lines at acidic pH. (**A**) *Dictyostelium* PsenA^-^/B^-^ cell lines rescued with catalytically active (psenB) or inactive (psenBDD) *Dictyostelium* presenilin proteins are able to fully restore development regardless of neutral (pH 7) or acidic (pH 5) pH. (**B**) *Dictyostelium* PsenA^-^/B^-^ cell lines rescued with catalytically active (PSEN1) or inactive (PSEN1DD) human PSEN1 are able to fully restore development regardless of neutral (pH 7) or acidic (pH 5). Data are representative of 3 independent experiments.


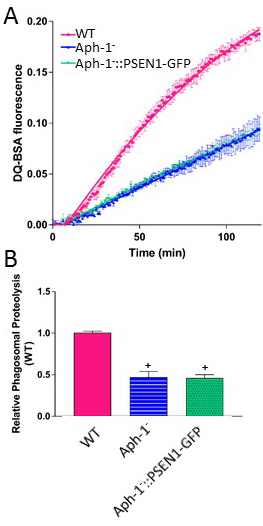


**Figure S6.** Acidification defects are shown in mutants lacking orthologous components of the γ-secretase complex. (**A**) Phagocytically engulfed DQ-BSA beads exhibit decreased fluorescence in Aph-1^-^ cells compared to wild-type cells (p<0.05). Expression of human PSEN1-GFP in Aph-1^-^ cells does not rescue this defect indicating this process is not driven by Presenilin alone in *Dictyostelium*. (**B**) Normalized phagosomal proteolysis demonstrates a ~60% decrease in proteolysis in Aph-1^-^ and Aph-1^-^::PSEN1-GFP cells when compared to wild-type cells. Data are derived from triplicate independent experiments, and ^+^ denotes significance against wild-type cells.


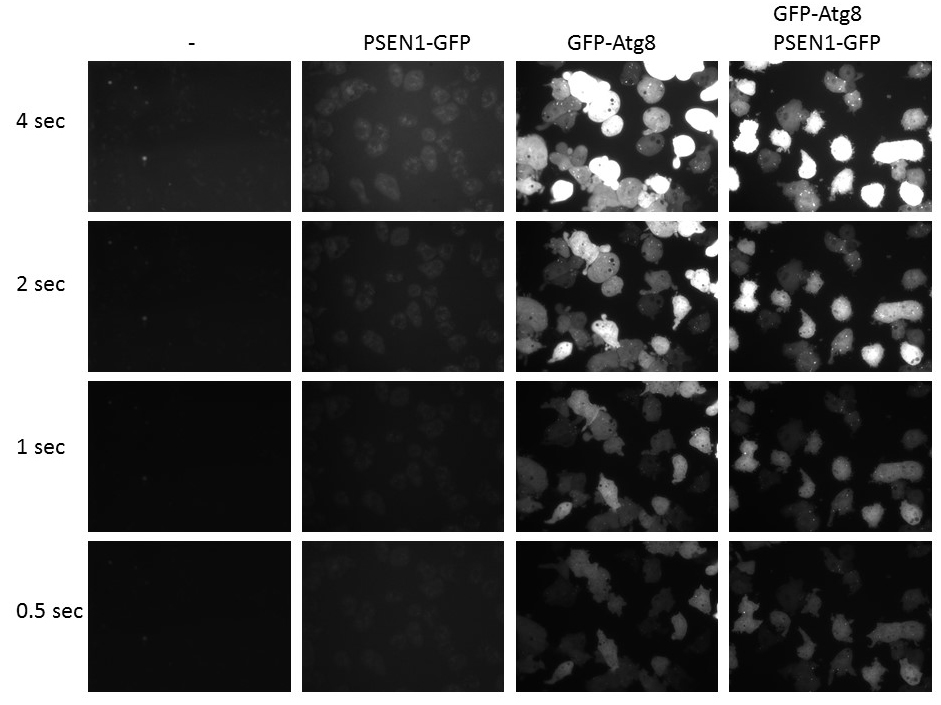


**Figure S7.** Comparative fluorescence of PSEN1-GFP and GFP-Atg8 in *Dictyostelium*. Wild-type cells (-), and cells expressing PSEN1-GFP, GFP-Atg8, and both PSEN1-GFP, GFP-Atg8 were imaged for GFP fluorescence at the indicated exposure times. Comparison of resulting fluorescence images indicates that under these conditions, the majority of fluorescence is derived from GFP-Atg8.


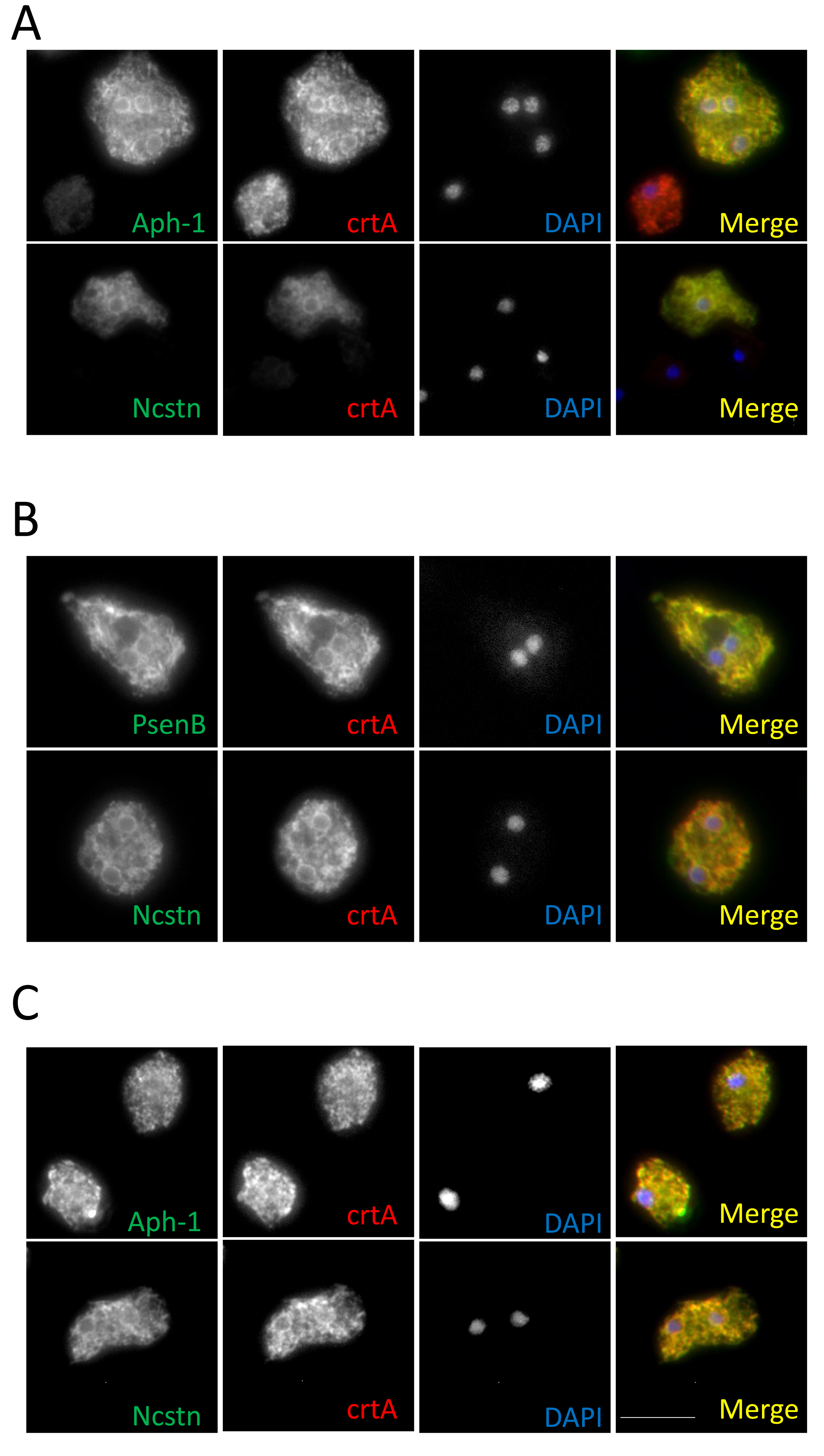


**Figure S8.** Localization of orthologous components of the γ-secretase complex in *Dictyostelium*. (**A**) Immunofluorescence for Aph-1-GFP and Ncstn-GFP in wild-type cells show endoplasmic reticulum localization, colocalizing with the ER marker crtA (calreticulin). (**B**) Localization of psenB-GFP and Ncstn-GFP in Aph-1^-^ cell lines still demonstrate colocalization with crtA indicating ER localization. (**C**) Localization of Aph-1-GFP and Ncstn-GFP in PsenA^‑^/B^-^ cells is unaltered. Size bar: 10 μm.

**
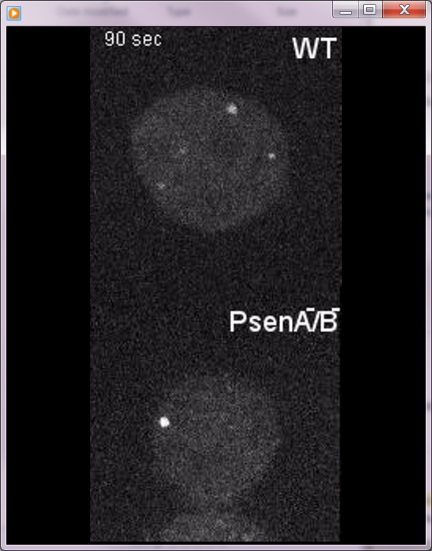
**

**Movie S1.** GFP-Atg8 Quenching in wild-type and PsenA^-^/B^-^ cells. A representative video comparison of the time taken for GFP-Atg8 to be quenched in wild-type and PsenA^-^/B^-^ cells. In wild-type cells the characteristic autophagosome ring formed at time point 78 s has its signal quenched at 112 s. In PsenA^-^/B^-^ cells the autophagosome ring formed at 94 s quenches at time point 154 s. The image provided here needs a link to the movie, provided in upload of paper to journal.
